# The effects of acupuncture on cognitive impairment of vascular dementia patients: Protocol for a systematic review and meta-analysis

**DOI:** 10.1097/MD.0000000000017648

**Published:** 2019-10-25

**Authors:** Yinshan Tang, Shujun Shao, You Zhou, Bing Xiong, Jin Cao, Zhigang Li, Jihong Wu, Chao Wang

**Affiliations:** a Department of Rehabilitation in Traditional Chinese Medicine, The Second Affiliated Hospital of Zhejiang University School of Medicine, Hangzhou; b School of Acupuncture-Moxibustion and Tuina, Beijing University of Chinese Medicine, Beijing, China; c Department of Psychiatry, Massachusetts General Hospital, Harvard Medical School, Boston, MA; d Department of Radiology, The Second Affiliated Hospital of Zhejiang University School of Medicine, Hangzhou, China.

**Keywords:** acupuncture, protocol, systematic-review, vascular dementia

## Abstract

**Background:**

Vascular dementia (VaD) is the second prevalent dementia worldwide attributable to cognitive impairments. Acupuncture has been applied in clinic as a therapeutic modality to treat VaD. This systematic review and meta-analysis aims to evaluate current evidence to explore the effectiveness and safety of acupuncture treatment to cognitive impairment of VaD.

**Methods:**

Randomized controlled trials will be searched restricted to their inception from January 1, 2000 to September 15, 2019. The following literature databases will be searched, including 4 English databases: PubMed, Excerpta Medica Database, the Cochrane Library, Medline, and 4 Chinese databases, namely the China National Knowledge Infrastructure Database, the Wanfang Database, the Chinese Scientific Journal Database, and the Chinese BioMedical Literature Database. After the selection and extraction of eligible studies, a meta-analysis will be undertaken to assess the efficacy and safety of acupuncture on VaD. The Review Manager Software V.5.3.5 will be employed for meta-analysis to assess the risk of bias, data synthesis, and subgroup analysis.

**Results:**

The systematic review and meta-analysis will be carried out to evaluate the efficacy and safety of acupuncture in the treatment of VaD, further provide an evidence-based synthesis for clinical and research applications.

**Conclusions:**

The summary of our systematic review will determine whether acupuncture intervention to VaD is safe and well-tolerated in global status.

## 1 Introduction

Vascular dementia (VaD) is a heterogeneous group of disorders which accompanied with various neuropathological manifestations caused by cerebrovascular disease. Accordingly, VaD is exponentially age-related, marked with progressive and irreversible cognitive decline. Currently, many countries are undergoing a process of demographic transition in which the rates of elderly individuals aged ≧60 accounts are increasing dramatically, consequently resulting in the high prevalence of chronic dementia medical conditions.^[1,2]^ As reported, there are 7.7 million new dementia diagnosis^[3]^ annually worldwide. The increasing incidence of dementia has become an urgent public concerning health issue worldwide, especially in developing countries,^[4]^ where approximately 46% of patients with dementia reside.^[5]^ It is supported by epidemiology findings that the financial burden of dementia in the United States has already surpassed that of cancer and cardio-disease.^[6]^ While the age-specific dementia incidence has declined in some developed countries, probably due to the reduction of vascular disease as medical treatment advances,^[7,8]^ some people are still susceptible to dementia owing to increased life expectancy.^[9]^ Diabetes, hypertension, and dyslipidemia are all potential factors for increased risk of VaD.^[10]^ Level of education could also serve as an alternative effective indicator of cognitive reserves, which has a significant association with the progression and severity of VaD.

VaD can coexist with multiple cerebral vascular diseases that can affect cognition in the elderly, characterized with an increased risk of cognitive deterioration and hippocampal atrophy.^[11]^ Cerebral vessels also play vital role in transporting specific molecules between the brain and blood tissue, which are the leading component of blood-brain barrier (BBB). Multiple studies have reported that the increased BBB permeability in dementia contributes to neurodegeneration and cognition decline.^[12,13]^ Small vessel disease has been a common cause of VaD, but the underlying pathogenesis is poorly understood.^[14]^ Given these circumstance, some research have begun considering VaD as an extension of vascular disease, and sought to develop novel strategies that focus on blood vessel. It has been hypothesized that dysfunction of tight junction among capillaries resulting in BBB dysfunction and lead to dementia.^[15]^ Drugs like memantine and cholinesterase inhibition have been proven significantly effective in treating Alzheimer's disease (AD), and; therefore, are labeled for this indication; however, they are not recommended for the treatment of VaD either regulate bodies or guideline shops due to their overall low effectiveness and possible side effects.^[16]^

Acupuncture, recognized as an ancient Chinese medical procedures with minimal side effects, has been applied regularly in clinic for thousands of years in China, and is getting increasing interests worldwide these years. Acupuncture is a nondrug technique accomplished by manual manipulation or electrical stimulus through specific acupoints, which are located on meridians or collaterals. Accumulating studies demonstrated that traditional Chinese medicine (TCM) has abundant experience in treating vascular cognitive impairments with acupuncture,^[17–20]^ that are proved to be effective in neuroprotection and improve long-term potentiation (LTP) impairments. Despite much scientific progress in research of VaD with acupuncture treatment during the past few decades,^[16]^ the mechanism and therapeutic effects of acupuncture still remains equivocal.

The present meta-analysis will serve as an overall guide for clinical practitioners to gain a better understanding of treating and preventing VaD with acupuncture.

## 2 Methods

The protocol of the systematic review and meta-analysis has been registered in the International Prospective Register of Systematic Review (PROSPERO), the registration number is CRD42019141429. The further amendments and rationales will be tracked in the PROSPERO. The protocol was complied with standard guidelines of preferred reporting items for systematic reviews and meta-analyses protocols (PRISMA-P).

### 2.1 Eligible criteria for including studies

#### 2.1.1 Types of studies

We will only include the random controlled trials for data synthesis, among which the randomization methods, eligible diagnosis, eligible outcome measurement are clearly claimed. In this review, both parallel and crossover studies are eligible selection, but in crossover studies, only the first-phase data will be analyzed to calculate the effect size. While other trials like case report, or without established international standard diagnosis will be ruled out.

#### 2.1.2 Types of participants

We will include participants who are diagnosed with VaD, irrespective of the gender, age, disease duration or it's severity. Individuals that are suffering from cognitive impairments but caused by AD, Parkinson's disease, or dementia with Lewy bodies will be excluded. Participants undergoing cerebral vascular diseases but have not yet developed to dementia will also be ruled out.

#### 2.1.3 Types of intervention

##### 2.1.3.1 Experimental interventions

Any penetrating acupuncture that simulate specific acupoints will be included in the review study, such as manual acupuncture, electroacupuncture, scalp acupuncture, abdominal acupuncture. Trials that compare acupuncture plus another typical treatment with that treatment alone will also be included. While nonpenetrating acupuncture-like moxibustion, laser acupuncture, acupressure, or percutaneous electrical nerve stimulation, will be excluded. Trials that compare the effectiveness of different forms of acupuncture will also be ruled out. No limitation will be set on the duration of needles retaining, electrical stimulus frequency and treatment course.

##### 2.1.3.2 Control interventions

Either sham or placebo acupuncture will be considered as the control interventions. Meanwhile, we will also include groups that receive no active treatment, herbal medication/drugs or physical/mental training therapy.

#### 2.1.4 Types of outcome measures

##### 2.1.4.1 Primary outcomes

Primary outcomes will be determined by clinical psychological or physical improvement that measured by validated scales such as the mini-mental state examination, Hastgawa dementia scale, blessed dementia scale, activity of daily living. We will also include the imaging examination like cerebral computed tomography or magnetic resonance imaging which shows the level of amelioration or severity of atrophy degree.

##### 2.1.4.2 Secondary outcomes

Secondary outcomes will be measured by the incidence of any complications related to acupuncture intervention such as infection/inflammation around needling site, faint or dizziness during acupuncture intervention, discomfort after acupuncture will also be tracked. Meanwhile, the quality of life (QOL) will also be employed to evaluate the secondary outcomes.

### 2.2 Search methods for the identification of studies

#### 2.2.1 Electronic searches

The following 8 databases including PubMed, Excerpta Medica Database, the Cochrane Library, Medline, China National Knowledge Infrastructure, the Wanfang Database, Chinese Scientific Journal Database, and Chinese BioMedical Literature Database will be investigated from their inception to September, 2019. The search strategy is consisted of 3 parts: disease, intervention and study design: (“Vascular dementia” or “Vascular” or “dementia” or “Subcortical vascular” or “Arteriosclerotic dementia” or “Binswanger disease” or “Subcortical encephalopathy” or “Chronic progressive” or “Cognitive impairments” or “Cognitive deficits”) and (“Acupuncture” or “Acupuncture therapy” or “Acupuncture treatment” or “Manual acupuncture” or “Electroacupuncture” or “Scalp acupuncture” or “Abdominal acupuncture” or “Warm acupuncture” or “Needling” or “Needles” or “Acupuncture points”) and (“Randomized controlled trial” or “Controlled clinical trial” or “Randomized” or “Randomly” or “Placebo” or “Trial”). The details for searching PubMed is provided in Table 1. Trials that do not meet the inclusion criteria will not be included. The search strategy will also be applied to other electronic databases. An equivalent translation will be adopted for Chinese databases searching.

**Table 1:**
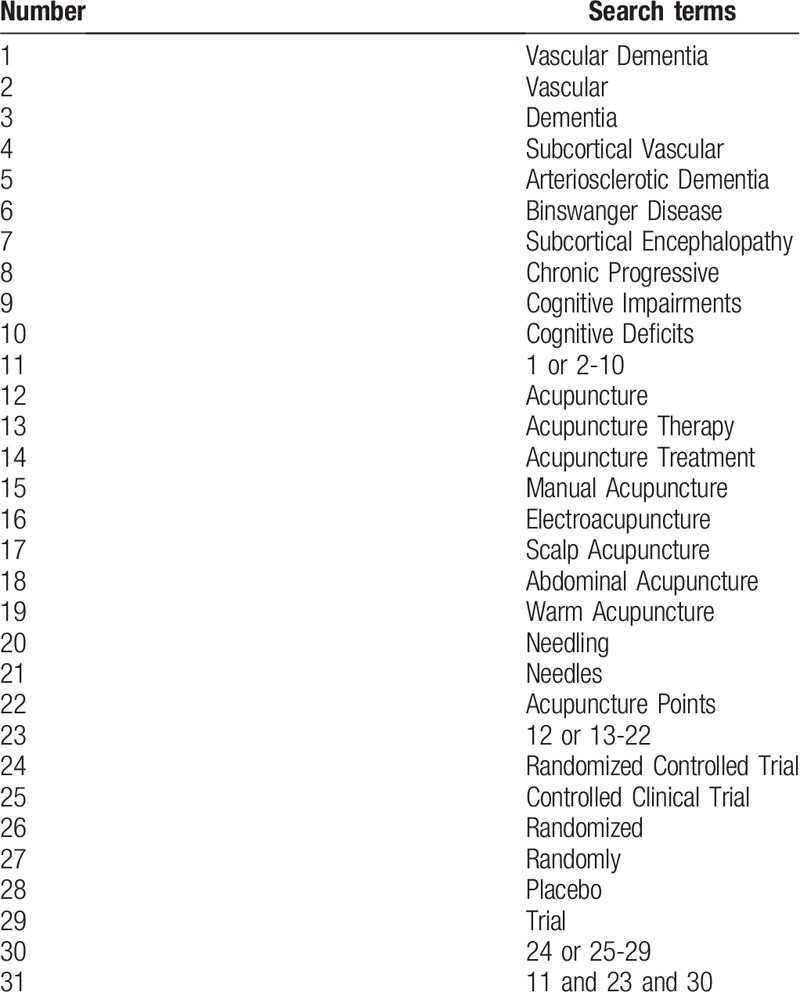
The search strategy performed in PubMed database.

#### 2.2.2 Search for other resources

Other electronic sources of trial registries including the WHO International Clinical Trial Registry Platform and Current Controlled Trials for ongoing and unpublished studies will also be searched to obtain relevant reports about VaD clinical treatment.

### 2.3 Data collection and analysis

#### 2.3.1 Study selection

**Figure 1:**
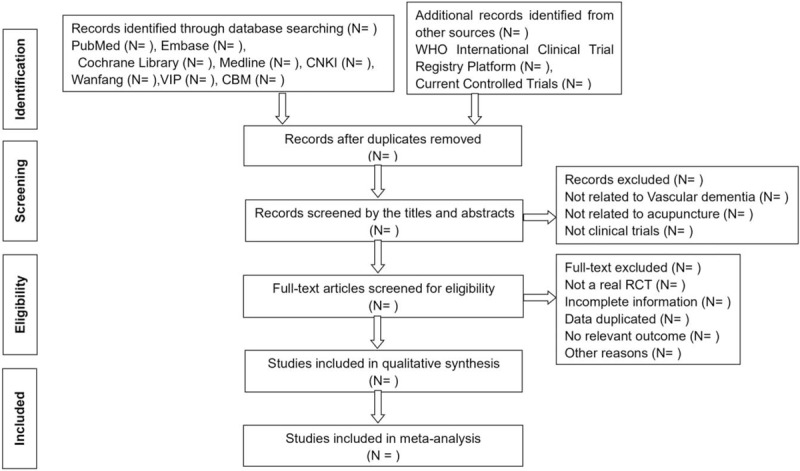
Preferred reporting items for systematic reviews and meta-analysis (PRISMA) flow diagram.

Before the conduction of selecting eligible studies, all the authors must get trained to ensure better understanding of the purpose and process of this review. Two reviewers (Yinshan Tang and Shujun Shao) will independently screen the titles, abstracts, and keywords for eligible studies, and subsequently exclude duplicate records. Then further assessment will be performed by reviewing the full-text, followed by the establishment of a table named “the reason for moved studies.” Any discrepancy between the 2 reviewers will be solved by plenary discussion till team consensus arrived. The selection procedure is fully described in a PRISMA flow chart (Fig. 1).

#### 2.3.2 Data extraction and management

Before the extraction of consistent studies, an electronic form will be made according to the inclusion criteria, filled by the 2 reviewers Yinshan Tang and Shujun Shao, who will independently extract studies based on the inception time, methodology, participants characteristics, intervention characteristics, outcomes, adverse effects, and other general information. Any disagreement or uncertainties will be resolved by consulting the arbiter (You Zhou). If needed, we will contact corresponding author for further detailed information of the trial.

#### 2.3.3 Assessment of risk of bias and quality of included studies

The 2 independent authors (Yinshan Tang and Shujun Shao) will execute the assessment of risk of bias (ROB) in each study based on the guidelines outlined in the Cochrane Handbook for Systematic Reviews of Interventions. The result of the assessment will also be crosschecked. Seven domains pertinent to ROB including generation of random sequence (selection bias), allocation concealment (selection bias), blinding of investigators and participants (performance bias), blinding of outcome assessment (measurement bias), dropout/loss of follow-up (attrition bias), selective outcome reporting (reporting bias), and other bias like conflicts of interest or follow up will be taken into full consideration for the final judgement. Each potential eligible study will be categorized or graded as low, unclear, and high ROB. Any discrepancy will be settled by the group discussion or adjudicated by an arbiter (You Zhou) to ensure the completion of the process.

#### 2.3.4 Measurements of treatment effect

For dichotomous data, the treatment effect size among studies will be presented as a risk ratio (RR) with 95% confidence intervals (CIs). Ordinal data will be converted to dichotomous data when data needs to be pooled. For example, different graded assessment like “recovery,” “remarkably effective,” “effective,” and “ineffective” will be pooled as “improved” or “not improved.” The mean difference (MD) with 95% CIs will be used to measure the treatment effect for continuous data.

#### 2.3.5 Unit of analysis issues

When the unit of analysis issues that assessed outcome variable repeatedly (more than 1 time point) arise in the conduction of research, we will focus on only 1 time point in the study.

#### 2.3.6 Dealing with missing data

For the missing or deformity data, we will attempt to contact the first author or corresponding author by e-mail or telephone for complete information. If we fail to obtain the missing data, adaptive selection and analysis will be relied on available data, and the potential influence of missing data will be addressed in the discussion section.

#### 2.3.7 Assessment of heterogeneity

Heterogeneity among the included studies will be assessed based on the guidelines of Review Manager 5.3.5 (RevMan 5.3.5) Software provided by the Cochrane Collaboration. The analysis of heterogeneity will be preferentially performed by the Chi-squared distribution and *I*^2^ statistic. When the *I*^2^ value is more than 50% and *P*-value is less than .10, we will indicate the heterogeneity among included manuscripts is significant large, and random effect model is adopted to analyze data and we will explore the source of the heterogeneity by conducting subgroup analysis or sensitive analysis. Otherwise, study will be considered no statistical heterogeneity, and we will employ fixed effect model to analyze the pooled data.

#### 2.3.8 Assessment of reporting bias

If more than 10 trials are included in the review, we will develop funnel plots to evaluate the reporting bias. When the funnel is symmetrical, it means no reporting bias existed, and dissymmetry indicates considerable reporting bias.

#### 2.3.9 Data synthesis and analysis

We will conduct statistical analysis using RevMan V.5.3.5 software for data synthesis. If *I*^2^ < 50%, a fix-effect model will be employed to evaluate MD and RR. Otherwise, the source of heterogeneity will be analyzed using a random-effect model to exclude obvious clinical heterogeneity.

#### 2.3.10 Subgroup analysis

If the data is substantial, we will carry out subgroup analysis to assess the specific influence of different intervention forms on pooled studies, including the acupuncture types (needles, stimulation intensity/frequency, treatment duration), the age/gender of participants, the severity of VaD, and other controlled interventions.

#### 2.3.11 Sensitivity analysis

Sensitivity analysis will be conducted to evaluate the robustness of the results by assessing the impact of sample size and methodological quality. Meanwhile, the effect of high attrition rates will also be taken into consideration to eliminate low-quality studies.

## 3 Discussion

Dementia is a syndrome manifested with general decline in cognition and memory function. VaD is the second prevalent type of dementia after AD, accounting for at least 20% cases of dementia. The first typical diagnostic criteria for VaD was devised in the early 1990s.^[21]^ The incidence rate of VaD annually has been reported to be 6-12 cases per 1000 individuals over 70 years old.^[22]^ Diverse types of risk factors have been reported, including advanced age, hypertension, high blood cholesterol, and arteriosclerosis^[23]^, arteriolosclerosis, cerebral infarcts, white matter changes, microbleeds, and dysfunction of cholinergic system,^[24]^ could cause moderate cerebral hypoperfusion and ischemic brain injuries in vulnerable regions of the brain, such as the hippocampus and cortex, leading to progressive cognitive decline and LTP impairments. To date, concurrently medication therapies are not satisfied, therefore, optional therapies are in urgent need for the treatment of VaD.

In TCM practice, acupuncture play a vital role in managing health condition, the therapeutic effects of which could exert through stimulating certain meridian acupoints. Multiple studies indicated that acupuncture could produce consistent and reproducible results in both clinical study and animal research for neural tissue recovery. In clinical study, acupuncture is becoming popular for treating cognitive impairments and dementia caused by vascular disease, which has been shown to lack adverse effects when compared with pharmaceutic therapies. It has been reported that stimulation with acupuncture could potentially excites nerve fibers, and improve the local blood circulation; therefore, stabilize and accelerate cerebral metabolic responses in multiple brain systems. In addition, animal research indicated that acupuncture could increase cerebral blood flow^[18]^ and restore LTP impairments^[25]^ to ameliorate dementia symptoms. However, the evidence of acupuncture-reduced cognitive improvements still need further elucidation to better understand the effects of acupuncture in different pathological states.

This review and meta-analysis will comprise RCTs in Chinese and English, which will provide reliable evidence that acupuncture may be considered as an effective intervention for the treatment of VaD and how it is shown to be superior to nonacupuncture therapies in relieving memory and cognition deficits of VaD patients. We hope this systematic review will provide a convincing conclusion on the effectiveness and safety of acupuncture on VaD for clinical practitioners, patients, and health policy conductors.

### 3.1 Ethics and dissemination

Formal ethics approval is not required in the protocol as we will conduct the work based on published articles. Since no patients are involved in this study, there will not be individual privacy concerning problems. We intend to explore the clinical efficacy of acupuncture to VaD, including the cognitive outcomes, QOL, better dig out the effectiveness and safety of acupuncture treatment. Consequently, we will submit the review results to a peer-reviewed journal or neurological related conference.

## Author contributions

**Conceptualization:** Yinshan Tang.

**Data collection:** Yinshan Tang, Shujun Shao.

**Formal analysis:** Yinshan Tang, Shujun Shao.

**Funding acquisition:** Yinshan Tang, You Zhou, Zhigang Li.

**Resources:** Yinshan Tang, Shujun Shao, Bing Xiong, Zhigang Li.

**Software:** Bing Xiong, Jin Cao, Zhigang Li, Jihong Wu, Chao Wang.

**Supervision:** You Zhou.

**Writing – original draft:** Yinshan Tang, Shujun Shao.

**Writing – review and editing:** Bing Xiong, Jin Cao, Zhigang Li, Jihong Wu, Chao Wang.

## References

[1] Nitrini R Bottino CM Albala C Prevalence of dementia in Latin America: a collaborative study of population-based cohorts. Int Psychogeriatr 2009;21:622 30. 19505354 10.1017/S1041610209009430 PMC8324310

[2] Kalaria RN Maestre GE Arizaga R Alzheimer's disease and vascular dementia in developing countries: Prevalence, management, and risk factors. Lancet Neurol 2008;7:812 26. 18667359 10.1016/S1474-4422(08)70169-8 PMC2860610

[3] Iadecola C The pathobiology of vascular dementia. Neuron 2013;80:844 66. 24267647 10.1016/j.neuron.2013.10.008 PMC3842016

[4] Lam T Sun K Chan H Perceptions of Chinese towards dementia in Hong Kong—diagnosis, symptoms and impacts. Int J Environ Res Public Health 2019;16:E128. 30621271 10.3390/ijerph16010128 PMC6339208

[5] Wimo A Winblad B Jönsson L An estimate of the total worldwide societal costs of dementia in 2005. Alzheimers Dement 2007;3:81 91. 19595921 10.1016/j.jalz.2007.02.001

[6] Hurd MD Martorell P Delavande A Monetary costs of dementia in the United States. N Engl J Med 2013;368:1326 34. 23550670 10.1056/NEJMsa1204629 PMC3959992

[7] Ahmadi-Abhari S Guzman-Castillo M Bandosz P Temporal trend in dementia incidence since 2002 and projections for prevalence in England and Wales to 2040: modelling study. BMJ 2017;358:j2856. 28679494 10.1136/bmj.j2856 PMC5497174

[8] Satizabal CL Beiser AS Chouraki V Incidence of dementia over three decades in the Framingham Heart Study. N Engl J Med 2016;375:523 32. 26863354 10.1056/NEJMoa1504327 PMC4943081

[9] Bennett JE Li G Foreman K The future of life expectancy and life expectancy inequalities in England and Wales: Bayesian spatiotemporal forecasting. Lancet 2015;386:163 70. 25935825 10.1016/S0140-6736(15)60296-3 PMC4502253

[10] Sahathevan R Brodtmann A Donnan GA Dementia, stroke, and vascular risk factors; a review. Int J Stroke 2015;7:61 73. 10.1111/j.1747-4949.2011.00731.x 22188853

[11] Gorelick PB Scuteri A Black SE Vascular contributions to cognitive impairment and dementia: a statement for healthcare professionals from the American Heart Association/American Stroke Association. Stroke 2011;42:2672 713. 21778438 10.1161/STR.0b013e3182299496 PMC3778669

[12] Zlokovic BV The blood-brain barrier in health and chronic neurodegenerative disorders. Neuron 2008;57:178 201. 18215617 10.1016/j.neuron.2008.01.003

[13] Schreiber S Bueche CZ Garz C Blood brain barrier breakdown as the starting point of cerebral small vessel disease? – new insights from a rat model. Exp Transl Stroke Med 2013;5:4. 23497521 10.1186/2040-7378-5-4 PMC3618264

[14] Wardlaw JM Smith C Dichgans M Small vessel disease: mechanisms and clinical implications. Lancet Neurol 2019;18:684 96. 31097385 10.1016/S1474-4422(19)30079-1

[15] Toyama K Spin JM Mogi M Therapeutic perspective on vascular cognitive impairment. Pharmacol Res 2019;146:104266. 31108183 10.1016/j.phrs.2019.104266

[16] O’Brien JT Thomas A Vascular dementia. Lancet 2015;386:1698 706. 26595643 10.1016/S0140-6736(15)00463-8

[17] Zhu W Wang XR Du SQ Anti-oxidative and anti-apoptotic effects of acupuncture: role of thioredoxin-1 in the hippocampus of vascular dementia rats. Neuroscience 2018;379:281 91. 29592844 10.1016/j.neuroscience.2018.03.029

[18] Su X Wu Z Mai F ‘Governor vessel-unblocking and mind-regulating’ acupuncture therapy ameliorates cognitive dysfunction in a rat model of middle cerebral artery occlusion. Int J Mol Med 2018;43:221 32. 30431067 10.3892/ijmm.2018.3981 PMC6257833

[19] Yang JW Wang XR Zhang M Acupuncture as a multifunctional neuroprotective therapy ameliorates cognitive impairment in a rat model of vascular dementia: A quantitative iTRAQ proteomics study. CNS Neurosci Ther 2018;24:1264 74. 30278105 10.1111/cns.13063 PMC6490085

[20] Du SQ Wang XR Zhu W Acupuncture inhibits TXNIP-associated oxidative stress and inflammation to attenuate cognitive impairment in vascular dementia rats. CNS Neurosci Ther 2018;24:39 46. 29110407 10.1111/cns.12773 PMC6489958

[21] Roman GC Tatemichi TK Erkinjuntti T Vascular dementia: diagnostic criteria for research studies: report of the NINDS-AIREN International Workshop. Neurology 1993;43:250 60. 8094895 10.1212/wnl.43.2.250

[22] Wm VDF Scheltens P Epidemiology and risk factors of dementia. J Neurol Neurosurg Psychiatry 2005;76 Suppl 5:v2 7. 16291918 10.1136/jnnp.2005.082867 PMC1765715

[23] Torre DL Jack C Cardiovascular risk factors promote brain hypoperfusion leading to cognitive decline and dementia. Cardiovasc Psychiatry Neurol 2012;2012:367516. 23243502 10.1155/2012/367516 PMC3518077

[24] Damodaran T Müller CP Hassan Z Chronic cerebral hypoperfusion-induced memory impairment and hippocampal long-term potentiation defificits are improved by cholinergic stimulation in rats. Pharmacol Rep 2019;71:443 8. 31003155 10.1016/j.pharep.2019.01.012

[25] Xiao LY Wang XR Yang JW Acupuncture prevents the impairment of hippocampal LTP through β1-AR in vascular dementia rats. Mol Neurobiol 2018;55:7677 90. 29435917 10.1007/s12035-018-0943-x

